# Black people are convicted more for being black than for being poor: The role of social norms and cultural prejudice on biased racial judgments

**DOI:** 10.1371/journal.pone.0222874

**Published:** 2019-09-20

**Authors:** Tiago Jessé Souza de Lima, Cicero Roberto Pereira, Ana Raquel Rosas Torres, Luana Elayne Cunha de Souza, Iara Maribondo Albuquerque

**Affiliations:** 1 Department of Psychology, Universidade de Fortaleza, Fortaleza, Ceará, Brazil; 2 Department of Psychology, Universidade Federal da Paraíba, João Pessoa, Paraíba, Brazil; 3 Institute of Social Sciences, Universidade de Lisboa, Lisboa, Portugal; Mälardalen University, SWEDEN

## Abstract

Black and poor people are more frequently convicted of committing crimes. However, the specific role played by skin color and social class in convicting a person has yet to be clarified. This article aims to elucidate this issue by proposing that belonging to a lower social class facilitates the conviction of black targets and that this phenomenon is because information about social class dissimulates racial bias. Study 1 (N = 160) demonstrated that information about belonging to the lower classes increases agreement with a criminal suspect being sentenced to prison only when described as being black. Furthermore, Studies 2 (N = 170) and 3 (N = 174) show that the anti-prejudice norm inhibits discrimination against the black target when participants were asked to express individual racial prejudice, but not when they expressed cultural racial prejudice. Finally, Study 4 (N = 134) demonstrated that lower-class black targets were discriminated against to a greater degree when participants expressed either individual or cultural prejudice and showed that this occurs when racial and class anti-prejudice norms are salient. The results suggest that social class negatively affects judgments of black targets because judgment based on lower class mitigates the racist motivation of discrimination.

## Introduction

Black and poor people are disproportionately more convicted of crimes for which judges assign longer sentences [1] so they are strikingly overrepresented in the prison population. For instance, in the USA, black Americans are incarcerated at a rate of more than five times that of whites [2]. In Portugal, in which this study was carried out, this scenario is no different. Due to its colonial past, Portugal has historically been a destination for immigration of black people mainly from the former Portuguese-Speaking African Colonies (PSAC). Although predominates in the Portuguese imaginary the luso-tropicalism ideology, referring to the idea of a supposed Portuguese ability for biological and cultural miscegenation with the peoples from tropics constituting a harmonious multiracial society [3], Portugal is not an exception in the scenario of racial attitudes and behaviors. Data from 2016 show that, proportionally, the rate of incarceration among the PSAC immigrants and PSAC descendants is 10 times higher compared with Portuguese citizens [4]. In addition, the proportion of black and white incarceration in Portugal may be even more unequal, since the Portuguese Constitution prohibits collecting ethnic-racial data in official surveys.

A similar phenomenon occurs with people from low social class, with these people from lower classes being more incarcerated than people from the upper classes [5,6]. The disparity becomes more pronounced when the two categories are combined, revealing a racially based class disparity in imprisonment: black Americans from the low social classes are much more incarcerated than white people from any social class [7]. This is ubiquitous in the world [1,7], which clearly suggests that racial and class inequalities in the prison population are a pervasive phenomenon. However, this has not yet been satisfactorily addressed from a social psychological point of view. In fact, social class has received less attention in studies on discrimination, particularly in terms of its interaction with skin color [8], highlighting a gap in the prejudice-discrimination literature, which has only recently been addressed [9,10].

One possible explanation for race-based social class disparity in prisons might be merely formal. At first sight, black people from lower social classes would be more often convicted because they cannot afford good lawyers. That is, black individuals would be convicted at higher rates more for being poor than for being black. Indeed, although we find white and black people in all social classes, official data indicate there is a correlation between being black and belonging to more disadvantaged social classes [11]. In light of this social reality, it is common for individuals to associate characteristics related to the lower social class with stereotypes about blacks [10,12]. This effect is in line with what Jones [13] claimed in his classic textbook *Prejudice and Racism*: “one of the big difficulties we have is disentangling race from class, given that (…) blacks, in particular, and ethnic minorities in general, are found disproportionately in the lower economic strata” (p. 441).

This difficulty seems to be more prominent in the context of racially based class disparity when convicting individuals accused of crimes. For instance, if both categories—skin color and social class—exert an influence independent of the other [14], then one would find similar proportions of black and white individuals from lower social classes in the prison population, which is not the case [7]. Since lower-class black people are proportionally more incarcerated than white ones from the same social class, it is very probable that skin color is a primary factor in convicting decisions, which can indicate that black people are convicted more for being black than for being poor. Thus, it necessary to take into account together information about social class and skin color in the process of making a decision [10,15,16], especially about convicting individuals accused of crimes.

The current work presents a research program with the objective of better understanding the effects of skin color and socioeconomic class in convicting black and white individuals in a Portuguese context. Studies that have addressed the relationship between skin color and social class were conducted predominantly in an USA context [9,10], exposing a gap of studies about this subject in other contexts, such as in Portugal. In this sense, the present study aims to evaluate the effect of skin color and socioeconomic class in conviction in a previously under-researched cultural context within social psychology literature of prejudice and discrimination. There is no clear evidence to which extent psychological biases toward the effect of skin color and social class in conviction observed in USA context are the same observed in Portugal or in which extent they differ between these two contexts, since race relations in these two countries have historically occurred in different ways.

Despite these differences, in both contexts the proportion of convicted poor black people is much higher than that of their white counterparts, then information about social class is likely to affect black and white targets differently. This disproportion suggests that belonging to the lower social class facilitates the conviction of black defendants, increasing the disparity of color in prisons. Accordingly, we propose that information about belonging to a lower socioeconomic class negatively affects judgments of black people but not white people. This differential effect can occur because of at least two main reasons. First, it is already known that when more than one social category of a target is salient, people need to integrate multiple pieces of information to form an overall impression about the target, especially in the absence of any contextual dominance of one category over the other (i.e., the cross-categorization effect) [17]. The cross-categorization between skin color and socioeconomic class can create salient stereotyped information for a target in both dimensions (lower class black). In this case, the effects of each dimension are integrated [18], which means that people can discriminate more against a person who belongs to multiple disadvantaged out-groups (lower class black), as opposed to someone who belongs to a single out-group (black without information of their social class) or to someone who belongs to a positive in-group in a less-favored dimension (i.e., lower class white).

Second, given that the proportion of poor blacks convicted is much higher than that of whites, social class information is likely to affect blacks and whites differently. This disproportion suggests that the social class facilitates the conviction of blacks, increasing the disparity between black and whites in prisons. Thus, it is likely that belonging to the lower classes can facilitate discrimination against black people because it can mitigate the racial motivation to convict a black target. In this sense, prejudiced people can discriminate against a lower-class black target, using a non-racist justification, even in social contexts where the anti-prejudice norm prohibits racial discrimination [19,20]. Moreover, we go further by proposing that the race-based class disparity in prison sentences is motivated by cultural prejudice (stereotypes and prejudices that are culturally shared) amplifying the discrimination toward black people and lower classes, despite the anti-prejudice norm.

### Social norms and the cultural expression of prejudice

Social psychology research on prejudice and discrimination has shown that people take social norms into account when behaving towards minority groups. Social norms can be understood as rules that define patterns of thinking and acting that are appropriate or desirable for members of a group by prescribing attitudes and forms of social behavior that are structured by social values [21]. According to Sherif [21], individual ideologies and belief systems are based on the social norms of the groups with which the person identifies, such that individual views are largely a reflection of the group norms that have been internalized by the individual. The internalization of the anti-prejudice norm in the system of personal beliefs differentiates people with low and high prejudice [22], so that the internalization of the norm is associated with an internal motivation to respond without prejudice. On the other hand, people who have not internalized the anti-prejudice norm may seek to appear non-prejudiced, which does not reflect their internalized attitudes, but reveals a conformity to the social norm. There is as an external source of motivation to respond without prejudice [23,24], in a situation in which there is an information bias concerning both race and class targets [25,26].

Accordingly, because of the pressure of the anti-prejudice norm, individuals probably try to avoid supporting the conviction of a black person accused of crimes when the social context clearly proscribes expressing negative racial attitudes [27]. Instead, since individuals are motivated to appear as not prejudiced, they explicitly evaluate white targets more negatively than black targets when believing that prejudiced attitudes are being evaluated, while implicitly discriminating against blacks when they do not think that prejudiced attitudes are being evaluated [28]. In fact, research within the framework of the aversive racism theory [29] has demonstrated that prejudiced individuals avoid discriminating against black people in situations where recognition of prejudiced motivation for discrimination is obvious, but they still discriminate when it is socially appropriate, i.e., in situations in which normative responses are less clearly delineated insofar as they can be justified based on a factor other than race [30,31]. That is, prejudiced people behave in accordance with their prejudice in the presence of a facilitating factor for discrimination, focusing on a non-racial attribute to make biased judgments about other people [25].

In the current study we aimed to take a new look at the role played by social class in discrimination. We propose that information about low social class functions as a facilitating factor for convicting a black person, since the motivation for conviction can be attributed to a different factor than race. Indeed, maybe this is what motivates the idea that “black individuals are convicted more for being poor than for being black”. In other words, information about lower socioeconomic class facilitates the expression of racial prejudice and discriminatory behavior. If this is the case, then the selective use of the information about socioeconomic class to penalize a black target, but not a white target, should be influenced by racial prejudice since social class can mitigate normative pressure to avoid convicting a black target for racial reasons. This effect will be amplified especially in a situation where the anti-prejudice norm does not suppress it, which occurs when individuals exhibit cultural prejudice, that is, when people make evaluations based in negative cultural stereotypes associated with black people.

In fact, individuals are conscious of and understand the stereotypes and prejudices that are culturally shared [32]. According to Devine [33], prejudice is based on culture, i.e., it is socially created and shared among members of a particular group. Individuals within a society are conscious of prejudice against certain target groups, i.e., cultural prejudice against certain groups is salient and often internalized by the members of that society. For example, in a study conducted by Chateignier et al. [32], the participants were asked to respond to a scale on intelligence, motivation, and proficiency in the French language among students of Arab-French or French descent, based on both personal opinion and the opinion of French society. The results indicated that the participants were conscious of the negative cultural stereotypes associated with Arab-French students. However, when the students were asked to give their personal opinion, there were no differences between the characteristics attributed to the two groups. Camino, Silva, Machado and Pereira [34] obtained similar results in a study of racial prejudice in Brazil. In their study, the participants were asked to evaluate black and white people using adjectives, based on their personal opinion (individual prejudice) or the opinion of Brazilian society (cultural prejudice). When the participants were asked to make the evaluation based on their personal opinion, they attributed more positive and fewer negative adjectives to black people. However, when the answers were given based on the opinion of Brazilian society, the opposite pattern was observed, with more negative and fewer positive adjectives attributed to black people.

This effect occurred because the anti-prejudice norm influences the expression of prejudice at an individual level but does not exert pressure on the cultural expression of prejudice [35]. Thus, prejudice will be more easily expressed when people attribute this view to others. When they are focused on their own opinion, they tend to deny that they are prejudiced [34]. Accordingly, individuals only avoid expressing racial prejudice at an individual level and have no problem expressing it at a cultural level. According to our rationale, if the information about social class functions as a facilitating factor for convicting a black person even under the pressure of the anti-prejudice norm, then this facilitating effect will be amplified in the context of cultural prejudice where this normative pressure is attenuated. This is an important innovative aspect in the literature on prejudice and discrimination, since as far as we know, there are no experimental studies evaluating the influence of individual/cultural expression of prejudice on the race-based social class disparity in judgments of black and white targets regarding crimes.

## Overview of the studies

Over the course of four studies, we sought to evaluate whether information about belonging to the lower classes facilitates social support for convicting a black person of an offense and how individual/cultural prejudice and the anti-prejudice norm influence this phenomenon. In Study 1, we tested the hypothesis that information about belonging to the lower class affects judgments of black and white targets differently, leading to harsher judgments for the black target. In Studies 2 and 3, we attempted to replicate the previous study and advance the hypothesis by evaluating how the anti-prejudice norm affects the effect of information about belonging to the lower classes on judgments of black and white targets. Because the anti-prejudice norm suppresses expression of prejudice at an individual level, but not at a cultural one [33], Study 4 aimed to analyze the facilitating role played by being in a lower socioeconomic class in a situation where the pressure to suppress prejudiced attitudes is weaker, i.e., when people can freely express prejudice without being restricted by social norms. Those studies were carried out in accordance with the recommendations of the Conselho Nacional de Saúde (Brazil) and Instituto de Ciências Sociais da Universidade de Lisboa (Portugal). All subjects gave written informed consent in accordance with the Declaration of Helsinki. They were informed their answers are anonymous and cannot be linked to any personal information. Due to the nature of the data collected in these studies (database with aggregated information, with no possibility of individual identification), this research is exempt from appreciation by an ethics committee, according to Resolution 510/2016 of the Conselho Nacional de Saúde (Brazil).

## Study 1

Study 1 aimed to test the hypothesis that information about belonging to the lower socioeconomic classes increases agreement with the black target’s conviction, but not the white target’s conviction. The participants were presented with a scenario concerning the trial of a person who clearly committed a crime. This scenario provided them with information about the skin color (black vs. white) and socioeconomic class of the suspect (lower class vs. control). The participants’ task was to indicate their agreement with the conviction of the suspect. We elaborated this judgment-scenario in order to confront the participants with evidence of target guilt and the need to express an anti-prejudiced response. We predicted that if information about the social class plays a facilitating role in convicting blacks, then participants should agree more strongly with the conviction of a black target from the lower classes than of one without information about social class. We also predicted that information on social class should not facilitate the conviction of the white target because the situation does not put participants in the normative conflict between the evidence of target guilt and the need to appear unprejudiced when judging a white target.

### Methods

#### Participants and design

This study included the participation of 160 Portuguese university students from a university predominantly of white students (mean age of 23.5 years, *SD* = 5.9; and 65.6% male). The participants were randomly assigned to one of four conditions in a 2 (skin color: black or white) x 2 (socioeconomic class: lower or control) factorial design. A post-hoc sensitivity analysis [36,37] for main effects and interactions determined this research design and the sample size provided 80% power to detect an effect size of d = 0.44 or higher (equivalent to η^2^p = 0.046 for interaction effects) as calculated by WebPower [38].

#### Procedure

The participants were recruited and asked to collaborate in a study of people's opinions about everyday situations reported in major newspapers. The participants’ task was to read a newspaper article and respond to questions about the facts presented in the article (S1 Fig). One article showed the arrest of a man who had attempted to bribe a police officer to avoid a traffic ticket. In this scenario, the manipulation of information related to socioeconomic class occurred through indicating the characteristics of the car driven by the target, which could be either a Fiat Uno 45 (a common cheap and old car in Portugal used by very poor people), in the lower-class condition, or only the word “vehicle”, in the control condition. Access to a car, and more recently, the quality of the car, since ownership has become more widespread across society, has shown to be a reliable indicator of socioeconomic status [39], and has been used in other experiments in social psychology to manipulate social class [40]. The manipulation of skin color occurred through a photo of the target, which could be a photo of a white or black person.

#### Dependent variable

The participants were asked to answer a question regarding the extent to which they agreed that the suspect should be convicted and sentenced to prison for attempting to bribe the police officer. The response scale used to answer the question ranged from 1 (strongly disagree) to 7 (strongly agree). This variable was called the prison sentence.

#### Manipulation check

To test whether the manipulation used in the study was effective, the participants were required to report the target’s skin color and socioeconomic class at the end of the study. The manipulation of skin color was verified through a question, answered on a scale ranging from 1 (black) to 7 (white). The manipulation of socioeconomic class was verified through a question in which the participant reported the target’s socioeconomic class, answered on a scale ranging from 1 (low) to 7 (high). A *t*-test for independent samples showed that the manipulation of skin color was effective, *t*(158) = 37.3, *p* < .001, Cohen’s *d* = 5.89, with a mean of 6.47 (*SD* = 0.94) for the white condition and a mean of 1.38 (*SD* = 0.78) for the black condition. Another *t*-test for independent samples indicated that the manipulation of the socioeconomic class did not reach the *p* value cut-off point of .05, although it was very close, *t*(158) = 1.89, *p* = .06, Cohen’s *d* = 0.29; the mean for the lower-class condition was 3.75 (*SD* = 1.14), and the mean for the control condition was 4.08 (*SD* = 1.07). Importantly, for the lower-class condition, the mean was marginally significantly lower than the midpoint of the scale, *t*(70) = -1.87, *p* = .065, Cohen’s *d* = 0.44, which was not true for the control condition, *t*(88) = 0.69, *p* = .49, Cohen’s *d* = 0.14.

### Results

Initially, a 2 x 2 factorial analysis of variance (ANOVA) revealed a significant interaction between skin color and socioeconomic class for the prison sentence variable, *F*(1, 156) = 13.1, *p* < .001, η^2^_p_ = 0.08 (Cohen’s *d* = 0.59). The main effects of skin color, *F*(1, 156) = 1.45, *p* = .23, Cohen’s *d* = 0.19, and socioeconomic class, *F*(1, 156) = 0.04, *p* = .84, Cohen’s *d* = 0.0, were not significant.

**Fig 1:**
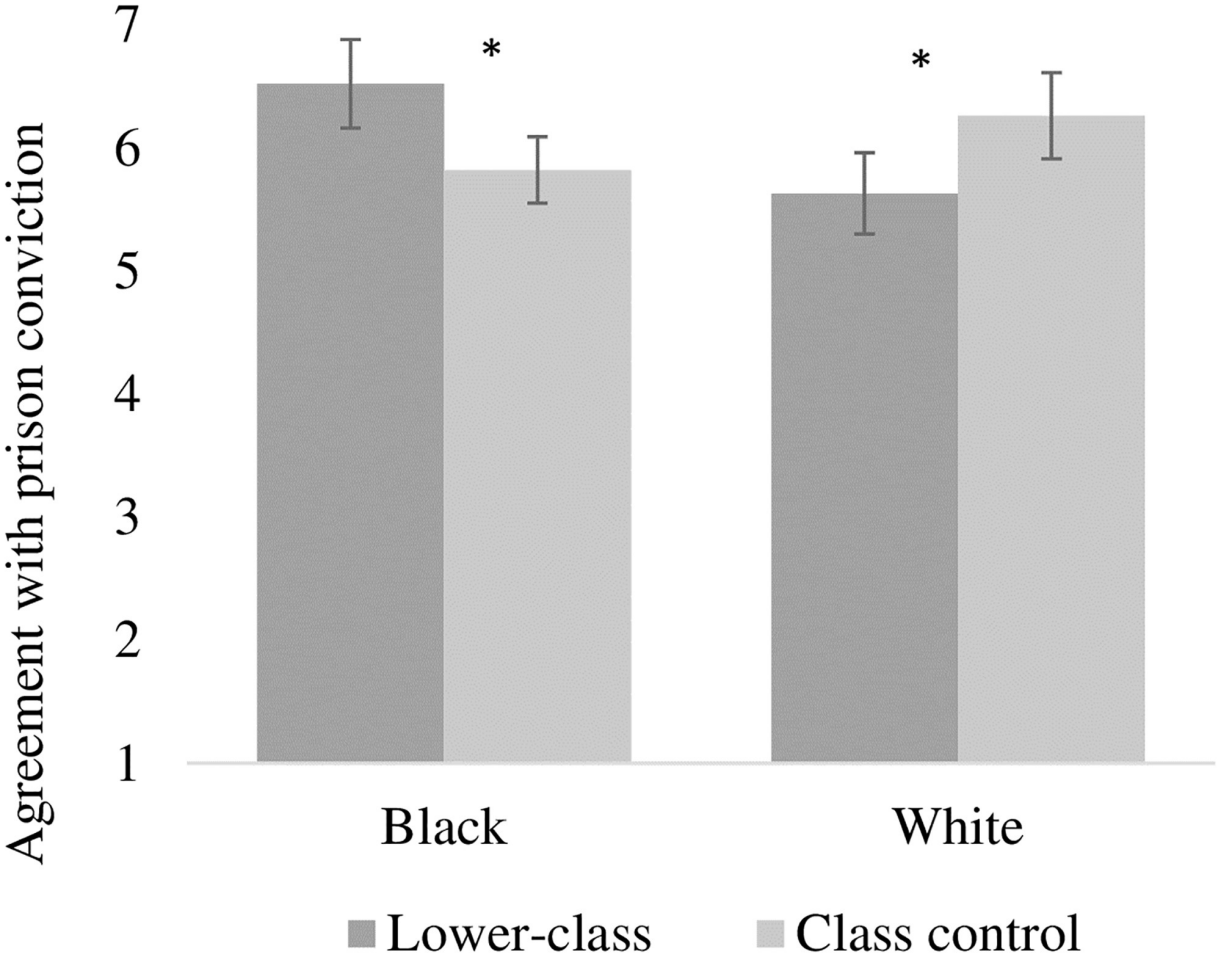
Agreement with prison conviction as a function of skin color and socioeconomic class.

Analyzing the effect of information about socioeconomic class on judgments of targets based on skin color, the pairwise comparisons indicated significant differences in the black and white conditions. For the black condition, agreement with the black target’s conviction was higher when he was described as lower-class (*M* = 6.52; *SE* = 0.20) than when there was no information about his social class (*M* = 5.82; *SE* = 0.15), *t*(156) = 2,34, *p <* .01, Cohen’s *d* = 0.37. For the white condition, agreement with conviction of the lower-class condition (*M* = 5.63; *SE* = 0.18) was lower than that for the control condition (*M* = 6.26; *SE* = 0.19), *t*(156) = 2.78, *p* < .01, Cohen’s *d* = 0.44. The means for the conditions are shown in Fig 1. Analyzing the interaction from another perspective, we found that support for the conviction of a lower-class black target was higher than that for a lower-class white target, *t*(156) = 3.26, *p* < .01, Cohen’s *d* = 0.52. When the participants had no information about social class, there was a tendency to favor the black target, with support for his conviction being lower than support for the conviction of the white target, *t*(156) = 1.79. *p* = .07, Cohen’s *d* = 0.28, although this effect wasn’t significant.

### Discussion

The results observed provide the first experimental evidence for the hypotheses that the information that a person who committed a crime belongs to a lower socioeconomic class increases support for the target conviction only for black targets, but not for whites. These results corroborate with other studies in the literature that have indicated the importance of considering information about more than one characteristic of an individual [18,41] when judging a particular target, in this case, socioeconomic class and skin color [14,42]. Results also indicate that individuals’ tend to differently consider information about belonging to the lower classes when judging blacks and whites.

One possible explanation for this effect is that when individuals are put in a motivational conflict between the evidence of guilt and the need to appear unprejudiced, they use information about belonging to the lower classes to mitigate the effect of the racial anti-prejudice norm [43]. In fact, participants showed a tendency to convict with lesser intensity the black target in a scenario without information about class, although the result (*p* = .07) did not reach the cut-off level of significance. This tendency probably reflects the effect of the anti-prejudice norm that is not observed in the condition in which there is information about belonging to the lower class. Furthermore, literature about the internal and external motivation to respond without prejudice determines that externally motivated individuals experience discomfort when judging situations related to race, encouraging a tendency to focus on attributes other than race, such as social attributes [23,26]. In this sense, Study 2 aims to evaluate the effect of social class more directly by reinforcing the salience of the anti-prejudice norm in judgments of black and white suspects.

## Study 2

This study aims to replicate the findings of the previous study and advance them, seeking to evaluate how the activation of the anti-prejudice norm influences the effect of information about belonging to the lower classes on judgments of black and white targets. Social norms such as the racial anti-prejudice norm directly influence the expression of prejudice and discrimination against social groups [44] because certain forms of prejudice are anti-normative or socially condemned [43]. In this study, the anti-prejudice norm is activated prior to the manipulation of skin color and socioeconomic class by giving clues to the participants that the study involved an analysis of racial issues. As in Study 1, we predicted that the participants would be more favorable to a conviction of a black suspect from a lower class than one without information about social class. Because individuals are motivated to appear unprejudiced when the anti-prejudice norm is present, we expected individuals to agree to a lesser extent with the black target's conviction, independent of any information about socioeconomic class. Indeed, the racial anti-prejudice norm leads people to avoid prejudiced personal attitudes [45] and does this by reinforcing positive attributes about devalued out-groups [28].

### Method

#### Participants and design

This study included the participation of 170 Portuguese students from a university predominantly of white students. However, four participants missed the manipulation check, so the final sample was 166 students, with a mean age of 20.6 years (*SD* = 2.43), the majority of whom were female (58.4%). Most of the participants reported that they were white (90.3%) and middle-class (72.3%). The participants were randomly assigned to one of eight conditions in a 2 (skin color: white or black) x 2 (socioeconomic class: lower or control) x 2 (anti-prejudice norm: salient or control) between-subjects factorial design. Post-hoc sensitivity analysis determined this research design and sample size provided 80% power to detect a medium effect size of d = 0.43 or higher (i.e., η^2^_p_ = 0.04 for interaction effects) as calculated by WebPower for main or interaction effects [38].

#### Procedure

We used a procedure similar to that used in Study 1 to manipulate the skin color and social class in which the task to be performed by the participants was to read a news item and answer some questions about the narrated facts. Since the manipulation of the socioeconomic class did not achieve a sufficient robust effect size in Study 1, some modifications were made to the manipulation used in this study. First, the news item used in this study is shorter and more accurate than that of the previous study, portraying a driver who runs over a pedestrian and drives away from the accident scene (S2 Fig). Second, the socioeconomic class was manipulated by giving information about the car driven by the target, as in Study 1, but we provided additional information about the characteristics of the car being in the lower-class condition (old, beat up car). Finally, the neighborhood where the target lived was also presented. So, in lower class condition, it was reported that the target was arrested at his home in a poor neighborhood. In the control condition, it was reported only that the target was arrested in his home, without giving information about the neighborhood.

The manipulation of the anti-prejudice norm was performed by presenting a racial prejudice scale prior to the manipulation (S1 Table). For the condition in which the norm was salient, the participants responded to a questionnaire about racial prejudice at the beginning of the study. When responding to questions about their prejudice, the participants would bear the norm in mind. For the control condition, the participants responded to racially neutral questions that addressed the importance of reading in the contemporary world. The racial prejudice questionnaire was expected to function as an activator of the anti-prejudice norm because it made information about skin color more salient and gave clues to the participants that the study might infer their level of prejudice. Furthermore, participants who indicated that they had taken part in similar previous studies were considered not eligible for this study.

#### Dependent variable

The dependent variable used was the same as that in the previous study. The participants indicated the extent to which they agreed that the suspect should be convicted and sentenced to prison. The response scale used to answer the question ranged from 1 (strongly disagree) to 7 (strongly agree).

#### Manipulation check

To verify the effectiveness of the manipulations, the participants reported the skin color and socioeconomic class of the article’s subject at the end of the study, using the same scales described in Study 1. Independent sample *t*-tests showed that the manipulations of skin color and social class were effective. The participants of the white condition reported a higher mean (*M* = 6.04; *SD* = 1.24) than those of the black condition (*M* = 1.54; *SD* = 0.76), *t*(164) = 27.73, *p* < .001, Cohen’s *d* = 4.37. Participants of the lower-class condition (*M* = 2.03; *SD* = 0.77) reported a lower mean than those of the class control condition (*M* = 3.74; *SD* = 0.77), *t*(164) = 14.06, *p* < .001, Cohen’s *d* = 2.22, indicating that the manipulation of the class was stronger and more effective than that used in Study 1. To verify the effectiveness of the norm’s activation, the participants answered a question on their opinion about the study’s real purpose. A chi-square test indicated a significant difference between the conditions, *χ*^2^ (3) = 67,7, *p* < 0,001. For the condition in which the anti-prejudice norm was active, 84.1% of the participants reported that the study was about racism, whereas only 13.6% of the participants in the control condition gave this answer. Therefore, this effect provided evidence that the salience of the anti-prejudice norm was effective.

### Results

A 2 (skin color: white or black) x 2 (socioeconomic class: lower or control) x 2 (anti-prejudice norm: salient or control) between-subjects factorial ANOVA indicated the main effect of skin color, *F*(1, 158) = 4.70, *p* < .05, Cohen’s *d* = 0.35, and the anti-prejudice norm, *F*(1, 158) = 4.77, *p* < .05, Cohen’s *d* = 0.35, are significant. The main effect of socioeconomic class was not significant, *F*(1, 158) = 0.06, *p* = .81, Cohen’s *d* = 0.04. Regarding the effect of skin color, the results indicated that agreement with the white target’s conviction (*M* = 5.80; *SE* = 0.17) was higher compared to the black target (*M* = 5.30; *SE* = 0.15). The effect of the anti-prejudice norm indicated that, in general, when the norm was active, agreement with the target’s conviction was lower (*M* = 5.30; *SE* = 0.16) compared to the condition when the norm was not active (*M* = 5.80; *SE* = 0.16). The non-significant effect of socioeconomic class may have occurred because manipulation of the social norm has made the skin color category more central to the social judgment.

**Fig 2:**
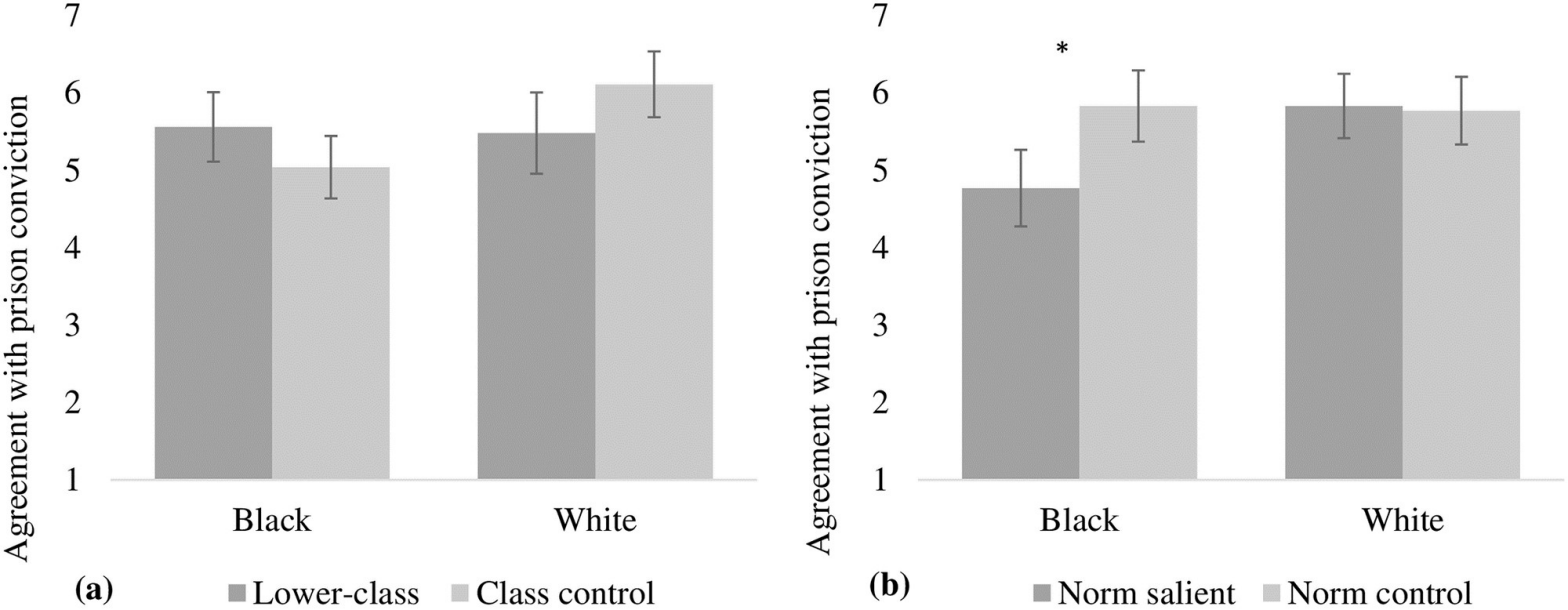
Agreement with prison conviction.

However, these main effects were qualified by interaction effects. A first interaction was observed between skin color and socioeconomic class, *F*(1, 158) = 6.27, *p* < .05, η^2^_p_ = 0.04 (Cohen’s *d* = 0.41). Although the mean difference did not reach the desirable level of significance using a two-tailed level of decision, pairwise comparisons indicated that participants have a tendency to agree more strongly with the conviction of a black target in the lower socioeconomic class condition (*M* = 5.56; *SE* = 0.23) than in the class control condition (*M* = 5.04; *SE* = 0.21), *t*(158) = 1.70, *p* = .091, Cohen’s *d* = 0.27. When the target was white, the participants have a tendency to agree less with their conviction in the lower-class condition (*M* = 5.48; *SE* = 0.27) than in the control condition (*M* = 6.11; *SE* = 0.21), *t*(158) = 1.83, *p* = .069, Cohen’s *d* = 0.29. These results are shown in Fig 2(a). It is possible that the smaller effect sizes observed have attenuated any significant differences and this might be due to the way the social norm was manipulated, since predominance of skin color was given in the categorization process.

A second interaction effect occurred between skin color and the anti-prejudice norm, *F*(1, 158) = 6.03, *p* < .05, η^2^_p_ = 0.04. The pairwise comparisons indicated that the black target's conviction was lower in the condition in which the norm was salient (*M* = 4.77; *SE* = 0.21) than in the condition in which the norm was not salient (*M* = 5.83; *SE* = 0.22), *t*(158) = 3.48, *p* < .01, Cohen’s *d* = 0.55. Regarding the white target, no significant differences were observed between the conditions in which the norm was salient (*M* = 5.83; *SE* = 0.25) and not salient (*M* = 5.77; *SE* = 0.23), *t*(158) = 0.18, *p* = .86, Cohen’s *d* = 0.03. These results are presented in Fig 2(b), which shows that the activation of the anti-prejudice norm inhibited the black target’s conviction.

The interaction between socioeconomic class and anti-prejudice norm was not significant, *F*(1, 158) = 0.01, *p* = .91, η^2^_p_ = 0.00. Also, the three-way interaction between color, class, and anti-prejudice norm was not reliable, *F*(1, 158) = 0.57, *p* = .45, η^2^_p_ = 0.00. Once again, it is possible that the absence of significant effects associated with social class is due to the predominance of skin color in the categorization process, which will be addressed in Study 4.

### Discussion

These results provided partial support for the hypothesis that information about belonging to the lower socioeconomic class harms blacks in situations involving social judgments. Moreover, this study also demonstrates that when the anti-racial prejudice norm is salient, participants tend to favor the black target. In this scenario, the participants tended to agree to a lesser extent with the black target’s conviction for the condition in which the norm was explicitly active compared to the condition in which the norm was not salient, which is in accordance with previous evidence that social norms are directly linked to the expression of prejudice and discrimination, and conforming to the norm leads to the suppression of prejudice and discrimination [45].

The pressure of the anti-prejudice norm causes people to avoid behaving in a way that could be assumed to be prejudiced. However, when individuals are in a situation in which prejudiced behavior is not likely to be credited to them, they can exhibit prejudice and discrimination against black people. Such a situation occurs when the individuals’ implication with the anti-prejudice norm was nullified, similar to what was observed by Camino et al [34], in a study in which the participants evaluated black and white targets according to personal opinion or society’s opinion. As noted previously, Devine [33] also made a distinction between individual and cultural prejudice. Accordingly, expressing individual prejudice is strongly suppressed by the anti-prejudice norm, while manifesting cultural prejudice is not, so individuals feel free to discriminate because they believe that their action will not be credited to their prejudiced motives. In the next study we analyze this possibility by asking the participants to judge the targets according to their own opinion (individual prejudice) and according to society’s opinion (cultural prejudice), in a context in which the anti-prejudice norm is explicitly active.

## Study 3

Previous studies have shown that information about belonging to the lower classes facilitates conviction of black targets but not white ones, however, in situations in which the anti-racial prejudice norm is explicitly salient, the participants tend to favor black targets, despite information about their socioeconomic class. What differentiates the individuals’ expression of a prejudiced or unprejudiced answer is the salience of the anti-prejudice norm [43], though prejudiced and unprejudiced individuals are conscious of culturally shared stereotypes and prejudices [33]. In a study conducted by Devine [33], the participants were asked to list stereotypes related to black people and were told that the study was interested in what society thinks about these stereotypes rather than in the participant's personal opinion. Results showed that participants exhibited blatantly negative stereotypes against black people at the cultural level, but not when asked to express their personal attitudes. That is, Devine’s [33] study developed an efficient experimental paradigm for suspending the pressure of the anti-prejudice norm to suppress prejudice responses.

Based on the paradigm developed by Devine [33], some studies have shown that people tend to freely express prejudice against blacks [34] and immigrants [32,35] when they respond on the behalf of society (cultural prejudice) but not when they respond for themselves (individual prejudice). According to Camino et al. [34] and Nunes [35], what explains these results is the fact that the anti-prejudice norm does not influence the expression of cultural prejudice, only the expression of individual prejudice. Individuals thus feel free to express prejudiced judgments insofar as they ascribe it to the culture and not to themselves.

Based on these findings, the current study aims to test whether the manipulation of prejudice (individual or cultural) affects judgments of targets based on their skin color and socioeconomic class when the anti-prejudice norm is clearly salient. So, we kept the anti-prejudice norm constant, while providing the participants with information about the race and social class of the target and manipulated the cultural prejudice (vs. individual). For the individual prejudice condition, we expected to observe results similar to those obtained in Study 2, i.e., lesser support for conviction of the black target than the white one, independent of information about social class. We further predicted that if using information about social class to convict the black target is motivated by prejudice, even in an anti-prejudice normative context, then the participants will agree to a greater extent with the lower-class black target’s conviction compared to the black target in the class control condition when they are asked to express cultural prejudice. On the other hand, information about belonging to the lower classes will not lead to greater discrimination against the white target in any of the prejudice conditions because information about belonging to lower social class to evaluate the white target is not motivated by prejudice, so convicting or not does not confront the anti-prejudice norm.

### Method

#### Participants and design

This study included the participation of 174 Portuguese students from a university predominantly of white students, with a mean age of 22.2 years (SD = 3.59), the majority of whom were female (53.4%). Most of them self-identified as white (95.3%) and middle-class (80.1%). The participants were randomly allocated to one of eight conditions in a 2 (skin color: white or black) x 2 (socioeconomic class: lower or control) x 2 (prejudice: individual or cultural) between-subjects factorial design. This research design and sample size provided 80% power to detect a medium effect size of d = 0.42 or higher (i.e., η^2^_p_ = 0.042 for interaction effects) as calculated by WebPower [38] for main and interaction effects.

#### Procedure

To make the anti-racial prejudice norm salient, a procedure similar to Study 2 was adopted. First, for all conditions, participants responded to a questionnaire about racism. The racial prejudice questionnaire was expected to function as an activator of the anti-prejudice norm because it made information about skin color more salient and gave clues to the participants so that the study might infer their level of prejudice. Then, we manipulated the skin color and social class by following the same procedure as Study 2. The manipulation of prejudice was done by adapting the procedure used by Devine [33] and previously used by Camino et al. [34], Fiske et al. [12] and Nunes [35] to manipulate cultural prejudice or stereotypes (S2 Table). The manipulation consisted of a response statement presented on the first page of the questionnaire. For the individual prejudice condition, the participants were instructed to respond based on their personal opinion. For the cultural prejudice condition, the participants were instructed to respond based on how society would respond. For this condition, it was reinforced that the objective was not to discover the participants’ personal opinion but rather what society thinks about the case. Furthermore, participants who indicated that they had taken part in similar previous studies were considered not eligible for this study.

#### Dependent variable

The dependent variable was the same as that used in Study 1. The participants indicated the extent to which they agreed that the suspect should be convicted and sentenced to prison. The response scale used to answer the question ranged from 1 (strongly disagree) to 7 (strongly agree).

#### Manipulation check

To verify the effectiveness of the manipulations, the participants were asked to indicate the skin color and socioeconomic class of the suspect presented in the article at the end of the study, using the same scales described in Study 1. Independent sample *t*-tests indicated that the manipulation of both skin color, *t*(153.6) = 35.6, *p* < .001, Cohen’s *d* = 5.31, and socioeconomic class, *t*(172) = 7.25, *p* < .001, Cohen’s *d* = 1.15, were effective. The mean reported by the participants for the white condition (*M* = 6.26; *SD* = 1.13) was higher than the mean for the black condition (*M* = 1.30; *SD* = 0.68). That is, the participants perceived the black target as actually black and the white target as really white. For the lower-class condition, the participants perceived the target as belonging to the lower class, given that the reported mean (*M* = 2.59; *SD* = 1.09) was significantly lower than that for the control condition (*M* = 3.74; *SD* = 0.90). To verify the effectiveness of the manipulation of prejudice, the participants responded to an adapted version of the Subtle and Blatant Prejudice Scale [22,46]. A *t*-test for independent samples confirmed the effectiveness of the manipulation used. The participants of the cultural prejudice condition (*M* = 3.41; *SD* = 1.37) presented a significantly higher mean than those of the individual prejudice condition (*M* = 1.91; *SD* = 1.03), *t*(153.9) = -8.04, *p* < .001, Cohen’s *d* = 1.22.

Regarding the activation of the anti-racial prejudice norm, the participants responded to the same question presented in the previous study. Approximately 85% of the participants reported that the study was about racism. Therefore, we believe that the activation of the anti-racial prejudice norm was effective.

### Results

A 2 (skin color: white or black) x 2 (socioeconomic class: lower or control) x 2 (prejudice: individual or cultural) between-subjects factorial ANOVA revealed a significant main effect of the manipulation of prejudice, *F*(1, 173) = 7.59, *p* < .01, Cohen’s *d* = 0.43. In general, the respondents presented a higher mean of agreement with conviction for the cultural prejudice condition (*M* = 5.76; *SE* = 0.13) than for the individual prejudice condition (*M* = 5.26; *SE* = 0.13). The main effects of skin color, *F*(1, 173) = 0.64, *p* = .42, Cohen’s *d* = 0.13, and socioeconomic class, *F*(1, 173) = 1.57, *p* = .21, Cohen’s *d* = 0.19, were not significant. Unlike Study 2, the main effect of skin color was not reliable, possibly due to the manipulation of prejudice (cultural vs. individual) introduced in this study.

**Fig 3:**
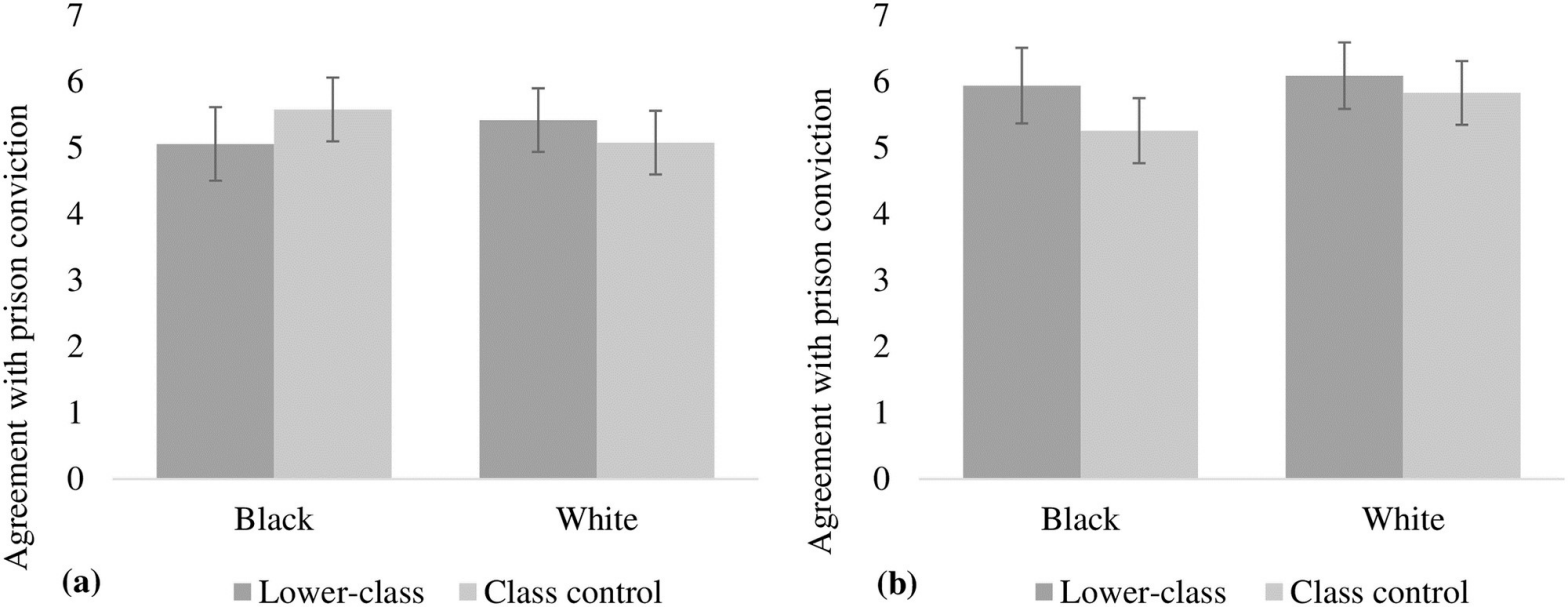
Agreement with prison conviction.

Importantly, we found a three-way interaction between skin color, class, and prejudice in the prison sentence, *F*(3, 173) = 2.92, *p* < .05, η^2^_p_ = 0.04 (Cohen’s *d* = 0.39). The decomposition of this interaction showed that the race*class two-way interaction for the individual prejudice condition was not significant, *F*(1, 173) = 1.28, *p* = .26, η^2^_p_ = 0.007. However, pairwise comparisons indicated that for the individual prejudice condition, no significant differences in agreement with the black targets’ conviction were observed between the lower class (*M* = 5.05, *SD* = 0.28) and control conditions (*M* = 5.58, *SD* = 0.24), *t*(173) = 1.43, *p* = .15, Cohen’s *d* = 0.22. That is, when the anti-prejudice norm was clearly reinforced, the information about social class did not facilitate conviction of the black target. For the condition in which the target was white, there were also no differences observed between the lower class (*M* = 5.42; *SE* = 0.24) and control conditions (*M* = 5.00; *SE* = 0.24), *t*(173) = 1.20, *p* = .23, Cohen’s *d* = 0.19. These results are shown in Fig 3(a).

For the cultural prejudice condition, the race*class interaction was also non-significant, *F*(1, 173) = 0.005, *p* = .94, η^2^_p_ = 0.00003. However, participants agreed strongly with the lower-class black target’s conviction (*M* = 5.94, *SE* = 0.29) compared to when there was no information about class (*M* = 5.18, *SE* = 0.25), *t*(173) = 1.98, *p* < .05, Cohen’s *d* = 0.79, i.e., the facilitating role played by lower social class was motivated by cultural prejudice. In the white condition, as expected, no significant differences were observed between the lower class (*M* = 6.09; *SE* = 0.25) and control conditions (*M* = 5.83; *SE* = 0.24), *t*(173) = 0.73, *p* = .46, Cohen’s *d* = 0.11. The results are shown in Fig 3(b).

### Discussion

The results obtained in this study clarify the findings of the previous study by showing that the conviction of black targets occurs when the participants are instructed to respond according to how society would respond. This effect is facilitated by information about the target belonging to the lower social classes. Furthermore, this study makes progress by demonstrating that even in a context in which the anti-prejudice norm is reinforced, cultural prejudice influences the evaluation of blacks and it is facilitated by information about the target’s socioeconomic class.

As in Study 2, this facilitating effect of social class disappeared when the anti-prejudice norm was clearly salient, such as what occurred for the individual prejudice condition in which, besides knowing that the study involved evaluating racial prejudice, participants were asked to express their personal attitudes. In fact, the main element that was highlighted in the condition of cultural prejudice was the absence of normative pressure for non-prejudice. For this reason, participants felt free to convict the black target, especially the poor one, even though they knew that the study was about prejudice.

Accordingly, the results of this study demonstrate that one of the reasons why cultural attributions occur is because they allow individuals to distance themselves from socially undesirable behaviors, offering a non-racial motivation for this behavior that serves as a protective factor for the ego [47]. According to our rationale, information about the lower socioeconomic class should contribute to this ego-protective effect because it is helpful to mitigate the prejudiced motivation in convicting the black target in the anti-prejudice normative context. This mitigating effect was not obtained from the individual prejudice condition, which means that the pressure exerted by the norm was so strong that it did not allow the social class information to have an effect. However, it is possible that the effect of socioeconomic class was weakened by the greater salience of the skin color category, when we made only the racial anti-prejudice norm salient, with little information given about the target’s class. It is possible that if both categories were equally salient in the context, the effect of the anti-prejudice norm could be attenuated by the information about socioeconomic class, allowing individuals to express their personal attitudes because social class provides them with a non-racial motivation for convicting the black target who clearly committed a crime. Study 4 seeks to address this possibility.

## Study 4

This study aims to replicate the racial-based class effect and develop its comprehension by analyzing whether reinforced information about social class facilitates the conviction of the black target even when the anti-prejudice norm is clearly salient. We specifically proposed that, when the participants have an equal quantity of information about race and class, they will agree to a greater extent with the conviction of the lower-class black target compared to the target with no information about class, for both the individual prejudice condition and the cultural prejudice condition.

### Method

#### Participants and design

This study included the participation of 134 Portuguese university students from a university predominantly of white students, with a mean age of 19.8 years (*SD* = 2.26), the majority of whom were female (63.4%). Most of the participants self-identified as white (90.3%) and middle-class (91%). The participants were randomly allocated to one of eight conditions in a 2 (skin color: white or black) x 2 (socioeconomic class: lower or control) x 2 (prejudice: individual or cultural) between-subjects factorial design. For all conditions, the anti-prejudice norms of color and class were salient. This research design and sample size provided 80% power to detect a medium effect size of d = 0.48 or higher (i.e., equivalent to η^2^_p_ = 0.05 for interaction effects) as calculated by WebPower [38].

#### Procedure

To make the norm salient, a procedure similar to that in Study 2 was adopted. Prior to the manipulation, the participants responded to a racial prejudice measurement (S1 Table) and a class prejudice measurement (S2 Table). We expected that the racial prejudice and class prejudice scales would function to activate the anti-prejudice norm because they made information about skin color and class more salient and gave clues to the participants in regard to the inference of their level of racism and classism. We reasoned that answering questions about attitudes to class would inhibit participants’ perception of the racial focus of the study, allowing information about social class to have more of an influence. Thus, we manipulated the skin color and social class of the targets by following the same procedure adopted in Study 2. The manipulation of anti-prejudice followed the same procedure as Study 3. Furthermore, participants who indicated that they had taken part in similar previous studies were considered not eligible for this study.

#### Dependent variable

The dependent variable was the same as that used in Study 1.

#### Manipulation check

The manipulation check was performed through questions about the suspect’s skin color and socioeconomic class, answered at the end of the questionnaire, using the same measurements described in Study 1. A *t*-test for independent samples showed that the manipulation of skin color was effective, *t*(113.5) = 25.5, *p* < .001, Cohen’s *d* = 4.47, and that the participants of the white condition (*M* = 6.13; *SD* = 1.21) reported a higher mean than those of the black condition (*M* = 1.38; *SD* = 0.90), indicating that the white target was perceived as white and the black target as black. Regarding the manipulation of socioeconomic class, a *t*-test for independent samples also indicated that the manipulation was effective, *t*(119.5) = 6.91, *p* < .001, Cohen’s *d* = 1.21, with the participants of the lower-class condition (*M* = 2.60; *SD* = 1.14) reporting a lower mean than those of the control condition (*M* = 3.80; *SD* = 0.79).

To check the effectiveness of the manipulation of prejudice, the participants responded to a racial prejudice scale and a class prejudice scale. These scales were adapted from the Subtle and Blatant Prejudice Scale [22,46]. The participants of the cultural racial prejudice condition (*M* = 3.62; *SD* = 1.5) presented a significantly higher mean than those of the individual racial prejudice condition (*M* = 2.24; *SD* = 0.99), *t*(118.7) = -6.20, *p* < .001, Cohen’s *d* = 1.07. The participants of the cultural class prejudice condition presented a significantly higher mean (*M* = 3.67; *SD* = 1.28) than those of the individual class prejudice condition (*M* = 2.60; *SD* = 0.97), *t*(132) = -5.46, *p* < .001, Cohen’s *d* = 0.95. Therefore, the manipulations of individual and cultural prejudice were effective.

### Results

A 2 (skin color: white or black) x 2 (socioeconomic class: lower or control) x 2 (prejudice: individual or cultural) between-subjects factorial ANOVA indicated significant main effects of skin color, *F*(1, 133) = 3.98, *p* < .05, Cohen’s *d* = 0.35, socioeconomic class, *F*(1, 133) = 8.24, *p* < .01, Cohen’s *d* = 0.50, and prejudice, *F*(1, 133) = 13.98, *p* < .001, Cohen’s *d* = 0.67. Regarding skin color, a higher agreement with the black target’s conviction (*M* = 5.66; *SE* = 0.13) was observed compared to the white target (*M* = 5.28; *SE* = 0.14). Concerning class, agreement with conviction for the lower-class condition (*M* = 5.74; *SE* = 0.13) was higher than for the control condition (*M* = 5.20; *SE* = 0.13). For prejudice, the respondents presented higher agreement with conviction for the cultural prejudice condition (*M* = 5.82; *SE* = 0.13) than for the individual prejudice condition (*M* = 5.11; *SE* = 0.14). However, these results were qualified by a significant two-way interaction between skin color and social class, *F*(1, 133) = 11,1, *p* < .01, η^2^_p_ = 0.08 (Cohen’s *d* = 0.59). Pairwise comparisons indicated that participants agree more strongly with the conviction of a black target of the lower socioeconomic class condition (*M* = 6.24; *SE* = 0.17) than with the class control condition (*M* = 5.07; *SE* = 0.19), *t*(126) = 4.49, *p* < .001. When the target was white, no differences were observed between lower-class condition (*M* = 5.24; *SE* = 0.20) and the control condition (*M* = 5.34; *SE* = 0.21), *t*(126) = 0.32, *p* = .75.

**Fig 4:**
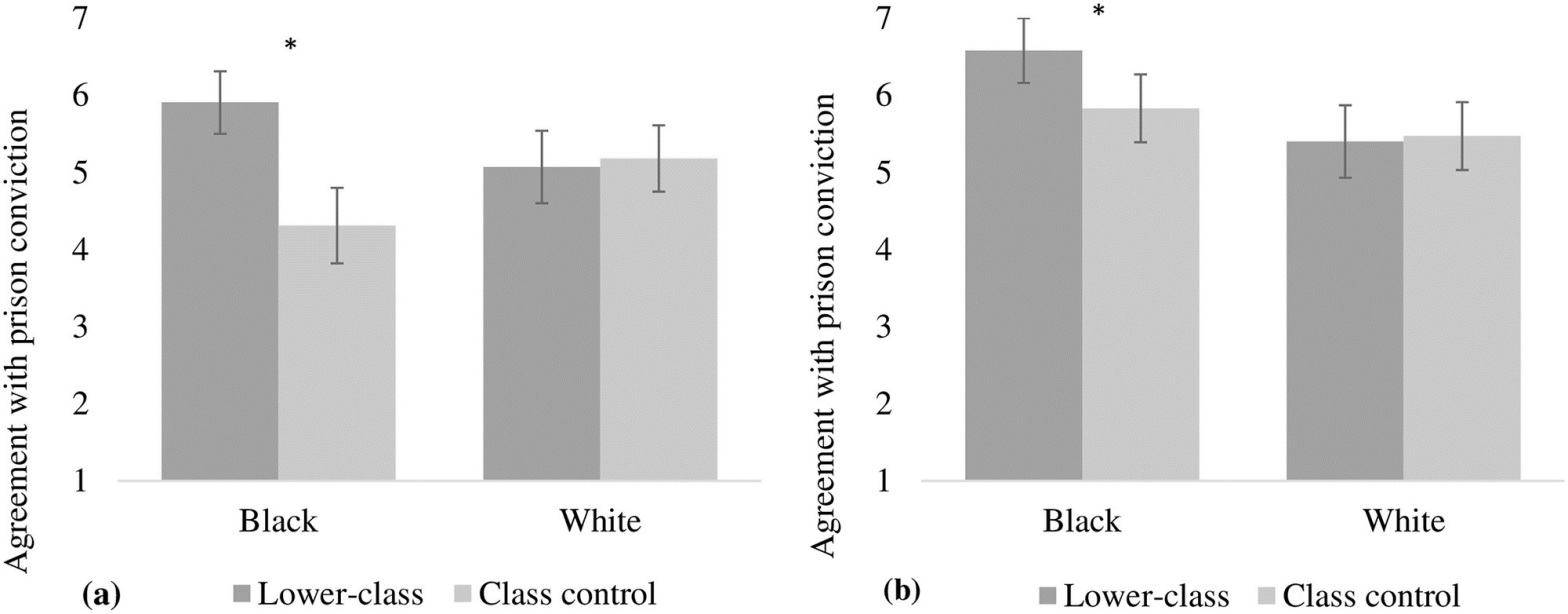
Agreement with prison conviction.

However, there was a three-way interaction between skin color, social class, and prejudice in the prison sentence, *F*(3, 126) = 2.16, *p* = .096, η^2^_p_ = 0.049 (Cohen’s *d* = 0.45), although not reaching the desirable level of significance, which would indicate significant differences between the conditions. The decomposition of this interaction showed a reliable race*class two-way interaction for the individual prejudice condition, *F*(1, 126) = 7.67, *p* < .01, η^2^_p_ = 0.056. For the individual prejudice condition, the planned comparisons indicated a significant difference in judgments of the targets of the black condition, *t*(126) = 4.19, *p* < .001, Cohen’s *d* = 0.74, with the participants agreeing to a greater extent with conviction of the lower-class condition (*M* = 5.91; *SE* = 0.24) compared to the control condition (*M* = 4.31; *SE* = 0.30). For the condition in which the target was white, no significant differences were observed, *t*(126) = 0.27, *p* = .78, Cohen’s *d* = 0.05, between the lower class (*M* = 5.07; *SE* = 0.29) and control conditions (*M* = 5.18; *SE* = 0.26). Results are presented in Fig 4(a), which shows that information about belonging to the lower social classes facilitated the conviction of the black target but not the white target.

Subsequently, for the cultural prejudice condition, the decomposition of this interaction showed a reliable race*class two-way interaction for the individual prejudice condition, *F*(1, 126) = 7.46, *p* < .01, η^2^_p_ = 0.055. Participants agreed to a greater extent with the black target’s conviction of the lower-class condition (*M* = 6.58; *SE* = 0.25) compared to the control condition (*M* = 5.83; *SE* = 0.25), *t*(126) = 2.09, *p* < .05, Cohen’s *d* = 0.37. For the condition in which the target was white, no significant differences were observed, *t*(126) = 0.18, *p* = .85, Cohen’s *d* = 0.03, between the lower class (*M* = 5.40; *SD* = 0.28) and control conditions (*M* = 5.47; *SD* = 0.26). These results are shown in Fig 4(b).

### Discussion

Results observed in this study provide supplementary evidence for our main hypothesis that information about social class facilitates conviction of black targets for a crime in the anti-prejudice normative context. In fact, in a context in which the racial and class information were equally salient, individuals agreed to a greater extent with convicting the lower-class black target, for both the individual prejudice and cultural prejudice conditions. Moreover, results provide further support for the prejudiced motivation for convicting the black target, since for the cultural prejudice condition this target was convicted to a greater extent than the white target. Results also replicate studies 1 and 2 in which the black target with no information about class was favored by the racial anti-prejudice norm for the individual prejudice condition. In other words, with this condition, individuals feel pressured to suppress discriminatory judgment to avoid violating the racial anti-prejudice norm [43]. However, when information about class is available and the class anti-prejudice norm is salient, individuals tend to convict the lower-class black target to a greater extent. For this condition, the pressure exerted by the racial anti-prejudice norm is mitigated, allowing participants to be free to convict a black target who clearly had committed a crime. Another aspect reinforcing this conclusion is the fact that information about class was used differently in judgments of white and black targets. If it were merely a matter of class prejudice, both white and black targets alike would be harmed. However, what we observed is that only black targets were harmed by this information, which leads us to believe that socioeconomic class serves as a facilitator of the expression of racial prejudice.

These results are even more compelling for the cultural prejudice condition. Although, for the individual prejudice condition, black targets (with no information about class) are protected by the norm, for the cultural condition, they are more discriminated against because individuals avoid responsibility for expressing racial prejudice individually but do not present a problem in expressing it at a cultural level, because they cannot be personally blamed for inflicting the anti-prejudice norm [34]. For this condition, information about belonging to the lower class facilitates discriminatory judgment because even when in a reinforced anti-prejudiced normative context, agreement with the black lower-class target’s conviction is higher than when there is no information about class. This pattern of results is in accordance with our prediction that convicting a black person from a lower social class in motivated by prejudice. This motivation influences individuals’ behavior when they feel free to judge a target believing that their judgment will not be attributed to racial motives.

## General discussion

Over the course of four experimental studies, we analyzed how belonging to the lower socioeconomic class affects judgments of black and white targets in a Portuguese context. In Study 1, we evaluated how information about belonging to the lower class directly affects judgments of white and black targets. In Study 2, we evaluated how the anti-prejudice norm affects the judgments of black and white targets from a lower-social class. Finally, in Studies 3 and 4 we manipulated prejudice (cultural vs. individual) to evaluate the effect of information about belonging to the lower class when the anti-prejudice norm is activated. Our results show that information about belonging to the lower class increases agreement with a prison sentence for the black target (Studies 1, 2, 3, and 4), while reducing (Studies 1 and 2) or not affecting (Studies 3 and 4) agreement with conviction for the white target. Furthermore, when the participants did not have information about the target’s class, they tended to favor the black target compared to the white target (Studies 1, 2, and 3). These results indicate that information about belonging to the lower classes facilitates convicting black targets.

The results also show that the racial anti-prejudice norm decreases agreement with the black target’s conviction (Studies 2 and 3). When we made both the racial and class anti-prejudice norms salient, agreement with the conviction of the lower-class black target was higher than that of the black target without any identified class (Study 4). We also observed that agreement with the conviction of the black target and lower-class black target is motivated by cultural prejudice (Studies 3 and 4).

### Theoretical implications

Although previous studies have demonstrated the importance of considering social class both independently of and in conjunction with skin color in a USA context [8,9], the results observed to date have not provided sufficient evidence in regard to what extent the effect of information about belonging to the lower classes has on judgments of black and white people [14,42,48], especially in the Portuguese context. The consistency of our results over four studies, in which different manipulations were performed, provides evidence for the hypothesis that belonging to the lower social classes will facilitate the conviction of the black target but not the white one.

The set of studies presented here also elucidates the effect of the anti-prejudice norm on the evaluation of targets belonging to more than a single social category (e.g., a lower-class black person), with one category being strongly protected by a norm [43]. When the racial anti-prejudice norm was salient, the participants tended to favor the black target, agreeing less with his conviction, compared to the condition in which the norm was not active (Studies 2 and 3). When skin color was the most salient category, the manipulation of the racial anti-prejudice norm was effective, leading to less agreement with the conviction of the black individual. Social norms are thus directly linked to the expression of prejudice and discrimination, suppressing the expression of prejudiced personal attitudes against groups protected by the norm [45].

However, although the anti-prejudice norm protects the black target, this effect occurs only in the absence of other factors than race to explain individuals’ behavior [27]. When other possible factors for discrimination exist (e.g., belonging a lower social class), individuals tend to convict the lower-class black target to a greater extent (Study 4). Although the manipulation we used for the racial anti-prejudice norm was effective, it made the skin color category more salient than socioeconomic class in Studies 2 and 3. Some studies on intergroup bias, in multiple categorization contexts, show that a number of factors can cause bias patterns that deviate from the frequently observed additive pattern (e.g., adding the effect of being lower-class to the effect of being black). In other words, increasing the salience of one of the categories or dimensions involved in the process can very often lead to the dominance of the category that is made more salient (e.g., predominance of categorization by color) [49]. In this sense, the activation of the racial anti-prejudice norm may have made the skin color category dominant in the judgment process, leading the participants to respond in a more egalitarian manner because the social context made clear that expressing negative racial attitudes is inappropriate.

Furthermore, this work provides evidence of individual and cultural expressions of prejudice in the Portuguese context. Our results show that when the anti-prejudice norm was active, the participants tended to favor the black target (with no information about class) compared to the white target, with regard to the expression of prejudice at an individual level (Studies 3 and 4). When they responded based on the judgments of society (cultural prejudice), this tendency disappeared, and the lower-class black target was convicted to a greater extent, suggesting that the facilitating effect of social class is motivated by racial prejudice.

The manipulation of the cultural or individual expression of prejudice provides results that are consistent with earlier studies on stereotypes about blacks and immigrants [32–34] and discrimination against immigrants [35] observed in other cultural contexts, like in France [32] and Brazil [34]. In our findings, the participants tended to favor the black target (with no information about class) when their judgments were based on individual opinion. When they made judgments based on Portuguese society’s opinion, they tended to convict the black target to a greater extent. This occurred because the racial anti-prejudice norm influences the expression of individual prejudice rather than the expression of cultural prejudice, i.e., the expression of cultural prejudice is not pressured by the anti-prejudice norm.

These findings also provide new insights concerning differences in the expression of individual and cultural prejudice. The cultural expression of prejudice allows for discrimination against the lower-class black target, even in a situation in which the norm is active and skin color is the most salient category (Study 3). However, in Study 4, in which we made equally salient both the race-based and class-based target categorizations prior to participants reading the scenario, we observed that the participants tended to convict the lower-class black target (but not the control) for both conditions—individual and cultural—though they convicted to a greater extent in the cultural condition.

The present research also advances prior investigations into the relationship between prejudice and discrimination. Indeed, the results indicate that information about belonging to the lower classes facilitates discrimination against black targets, in the expression of both individual and cultural prejudice. This proposition is also supported by our observations that belonging to the lower classes only harms the black target. The reason for this facilitation may be that the information about the socioeconomic class provided an unprejudiced reason for convicting a black target who clearly committed a crime. Specifically, it is possible that social class acts as a non-racist justification for discrimination in the Portuguese context, which is consistent with other studies that have shown that prejudice and discrimination against black people persist because people develop indirect ways to discriminate that do not confront the racial anti-prejudice norm [43]. The use of non-racist justifications to discriminate against black people is consistent with the myth of luso-tropicalism that permeates the vision of race relations in Portuguese society. If the Portuguese see themselves as an harmonious people seeking a benevolent and peaceful coexistence with people of different ethnic backgrounds [3,50], discrimination against black people will not occur on grounds that might be viewed as racist, but will be justified if the criteria to discriminate is seen as non-racist, like discrimination based on social class.

In fact, social status has long been used as a non-discriminatory justification for perpetuating or denying inequalities between whites and blacks. Many argue that “race” and ethnicity are not evaluative dimensions that are appropriate for addressing the problem of inequality because these differences are a product of socioeconomic disparities [51]. This possibility, based on beliefs in the integrative power of economic development and refined over several different iterations, is one of the pillars of the ideology of racial democracy, which is often evoked as an explanation of the undeniable inequalities between blacks and whites [52]. However, different studies have shown that “race” continues to have significant importance for the perpetuation of inequalities [52–54], and the results obtained here add to this evidence.

### Limitations and future directions

Although we present a set of four consistent studies, the results and conclusions obtained here so far are limited to the Portuguese context. Future research is needed to examine whether the results obtained here can be generalized to contexts with a history of racial relations closer to that observed in Portugal, as in Brazil, in which the ideology of racial democracy (similar to the ideology of Portuguese luso-tropicalism) prevails, characterized by a myth that blacks and whites live harmoniously in a multicultural society [55]; as well as in more well-studied social contexts, historically marked by segregated race relations, like the USA context.

Another limitation is the fact that we did not have a control for the effect of participants' skin color and socioeconomic class. Although the study was conducted with white participants and we randomly distributed the participants between the experimental conditions, it is possible that some observed effects, such as favoring the white target (with a higher status), were purely due to a tendency towards favoring the in-group [56]. Future studies with a more diverse sample are needed to address how differences in participants’ in-groups affect responses towards their out-groups. Another limitation is related to the manipulation of the anti-prejudice norm in Studies 2 and 3. In these studies, the manipulation of the racial anti-prejudice norm made the skin color category more salient than the social class category. It could thus be argued that the effect attributed to the anti-prejudice norm could be better explained by the salience of the category, i.e., the participants ignored socioeconomic class and focused only on the target’s skin color, leading to one category’s dominance over the other rather than the expected interaction effect [17]. Additionally, a pre-test was not performed on the photos used as stimuli to manipulate skin color. Some studies have indicated that features inferred from the face, such as trustworthiness and competence can influence social judgment [57,58]. Further research can counterbalance the salience of both categories and control the possible effect of trustworthiness and competence of the facial stimuli.

We also did not evaluate the stereotypical content of the subgroups used in our manipulations. Due to the important role of stereotypes, future studies should evaluate stereotypical content in multiple categorization contexts, helping to elucidate the similarities and differences between the class and skin color categories, and consequently whether the similarity in stereotypes shared by different groups (e.g., black people and poor people) are the basis of discrimination against this subgroup. Furthermore, elucidating stereotypes in this context can provide evidence for the additive effect of these two categories. We also did not consider other variables that could provide alternative explanations for our results. Other studies might test the role of system justification as a mediator between multiple categorizations and discrimination. Perhaps this mechanism can explain discrimination against low-status subgroups that have congruent stereotypes, perpetuating the existing social status quo. Future studies can also use the manipulation of individual and cultural prejudice to show differences between individual and social judgments in other contexts.

## Conclusions

In several cultural contexts across the world, the penal system has imprisoned more poor black than white people, independent of their social class. Analyzing this racially-based class disparity from a social-psychological point of view, our research provided sufficient experimental evidence for the hypothesis that white individuals have a tendency to support more easily the conviction of black people from lower social classes when they commit a crime. This tendency was not observed in judgment of white targets committing the same crime. Furthermore, this set of studies provides the first evidence regarding how the anti-prejudice norm and cultural and individual expressions of prejudice act to suppress or motivate the racially-based class disparity in sentencing a target to prison. When the racial anti-prejudice norm is salient, the black target is favored in individual but not in cultural judgments. Moreover, when the racial and the class anti-prejudice norm makes skin color and class salient, individuals discriminate against the lower-class black target to a greater extent, in both individual and cultural judgments.

## Supporting information

S1 Fig Manipulation of skin color and socioeconomic class—Study 1. (DOCX)

S2 Fig Manipulation of skin color and socioeconomic class—Studies 2, 3 and 4. (DOCX)

S1 Table Manipulation of individual and cultural prejudice. (DOCX)

S2 Table Manipulation of the racial anti-prejudice norm. (DOCX)

S3 Table Manipulation of the social class anti-prejudice norm. (DOCX)
