# Supplementary figures and images for: Black people are convicted more for being black than for being poor: The role of social norms and cultural prejudice on biased racial judgments
Source: PLoS One. 2019 Sep 20;14(9):e0222874. doi: 10.1371/journal.pone.0222874 (PMC6754140; doi:10.1371/journal.pone.0222874)

**S1 Fig. Manipulation of skin color and socioeconomic class – Study 1**

**
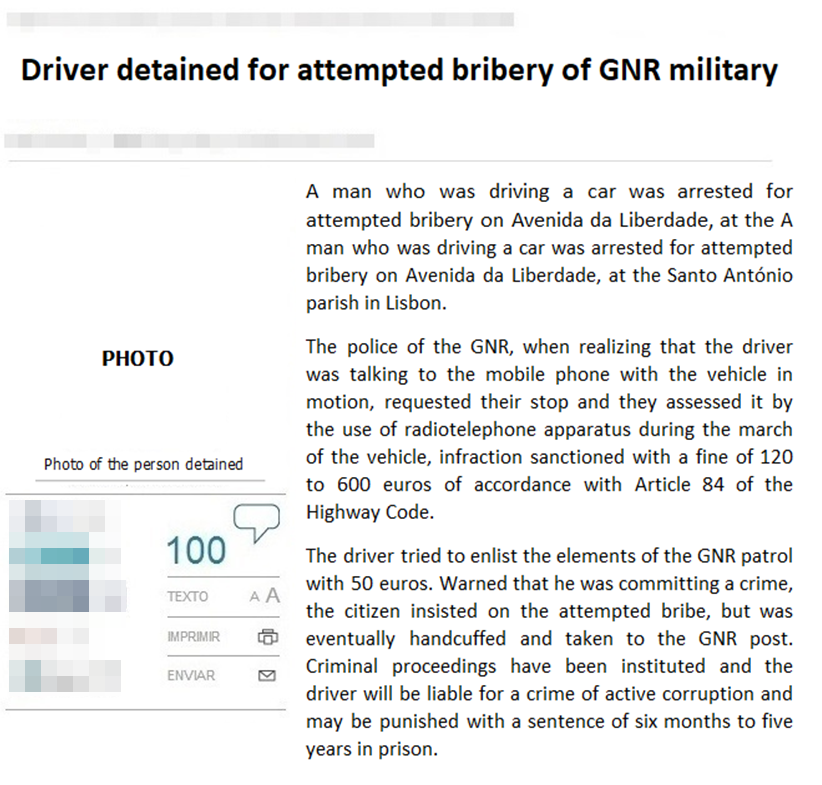
**

Supplement: S1 Fig — (DOCX) [file pone.0222874.s001.docx]

**S2 Fig. Manipulation of skin color and socioeconomic class – Studies 2, 3 and 4**

**
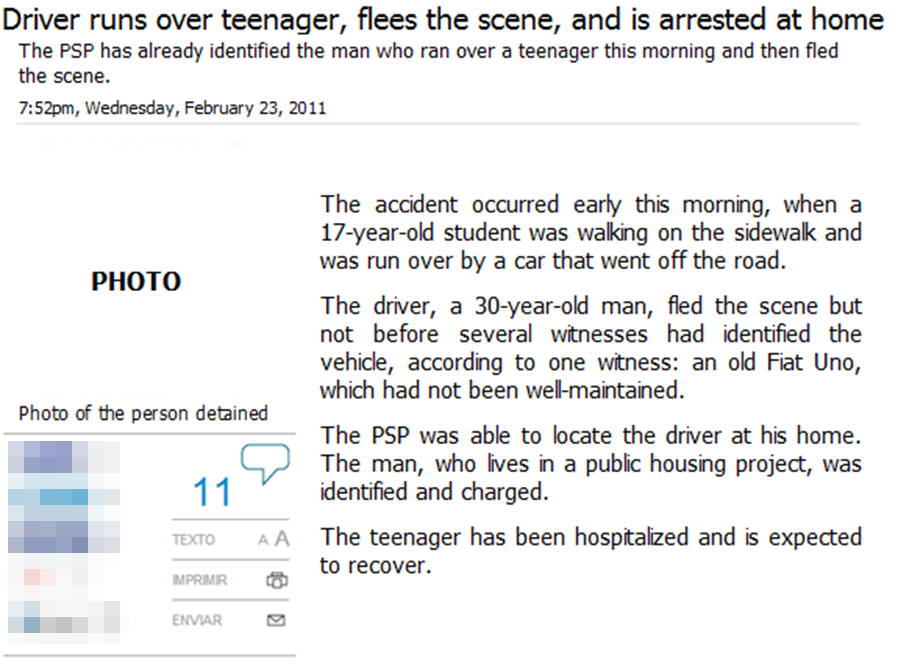
**

Supplement: S2 Fig — (DOCX) [file pone.0222874.s002.docx]
