# Supplementary material for: Black people are convicted more for being black than for being poor: The role of social norms and cultural prejudice on biased racial judgments
Source: PLoS One. 2019 Sep 20;14(9):e0222874. doi: 10.1371/journal.pone.0222874 (PMC6754140; doi:10.1371/journal.pone.0222874)
Supplement: S2 Table — (DOCX) [file pone.0222874.s004.docx]

**S3 Table. Manipulation of individual and cultural prejudice**

| *Cultural prejudice*  The following questions seek to understand what you imagine to be **the opinion of Portuguese society about the daily news published in newspapers**. It is important to note that **we do not want to know your personal opinion;** we want to know what you think **society thinks** about this news. |
| --- |
| *Individual prejudice*  The following questions seek to understand your opinion about the daily news published in newspapers. It is important to note that **we want to know your personal opinion** and not what society thinks about this news. |
