# Supplementary material for: Black people are convicted more for being black than for being poor: The role of social norms and cultural prejudice on biased racial judgments
Source: PLoS One. 2019 Sep 20;14(9):e0222874. doi: 10.1371/journal.pone.0222874 (PMC6754140; doi:10.1371/journal.pone.0222874)
Supplement: S3 Table — (DOCX) [file pone.0222874.s005.docx]

**S5 Table. Manipulation of the social class anti-prejudice norm**

Mark/reflect the extent to which you agree with the following ideas. Mark only one answer for each sentence; the higher the number, the more you think that society agrees with what was stated in the sentence.

| **Strongly Disagree** | **1** | **2** | **3** | **4** | **5** | **6** | **7** | **Strongly Agree** |
| --- | --- | --- | --- | --- | --- | --- | --- | --- |

| The poor belong to a less gifted class, and this explains why their situation is not as good as that of the rich. | **1** | **2** | **3** | **4** | **5** | **6** | **7** |
| --- | --- | --- | --- | --- | --- | --- | --- |
| The poor transmit values and skills to their children that are different from those needed to be successful in Portuguese society. | **1** | **2** | **3** | **4** | **5** | **6** | **7** |
| Compared to the rich, the poor teach their children very different values. | **1** | **2** | **3** | **4** | **5** | **6** | **7** |
| I would care if a close relative married a poor person. | **1** | **2** | **3** | **4** | **5** | **6** | **7** |
| Compared to the rich, the poor have very different values and social behaviors. | **1** | **2** | **3** | **4** | **5** | **6** | **7** |
| As far as honesty is concerned, the rich and poor are very different. | **1** | **2** | **3** | **4** | **5** | **6** | **7** |
